# Removing an Entrapped Pigtail Catheter by Re-enforcing a Traditional Method

**Published:** 2019-01

**Authors:** Alireza Serati, Babak Sharif-kashani, Zargham Hossein Ahmadi, Farah Naghashzadeh, Neda Behzadnia, Mandana Chitsazan, Payam Abbasi

**Affiliations:** 1 Chronic Respiratory Diseases Research Center, National Research Institute of Tuberculosis and Lung Diseases (NRITLD), Shahid Beheshti University of Medical Sciences, Tehran, Iran.; 2 Tobacco Prevention and Control Research Center, NRITLD, Shahid Beheshti University of Medical Sciences, Tehran, Iran.; 3 Lung Transplantation Research Center, NRITLD, Shahid Beheshti University of Medical Sciences, Tehran, Iran.

**Keywords:** Pulmonary artery catheterization, Cardiac catheter, Chordae tendineae

## Abstract

Right heart catheterization is the main step in the evaluation of pulmonary hypertension including Chronic Thromboembolic Pulmonary Hypertension (CTEPH) and is considered a relatively safe procedure. Complications can occur including perforation, tamponade, bleeding, etc. requiring different types of interventions such as manipulation or surgery. Here, we have described a case of pigtail catheter entrapment and the method we used to free it without invasive measures.

## INTRODUCTION

Chronic Thromboembolic Pulmonary Hypertension (CTEPH) is classified by the World Health Organization as group 4 Pulmonary Hypertension (PH) (1). This group is unique, as CTEPH is a form of pulmonary hypertension that is potentially curative without transplantation (2). CTEPH is caused by major vessel embolization, leading to pulmonary artery obstruction and remodeling (3). Right Heart Catheterization (RHC) is performed to confirm the diagnosis of PH (4). RHC is thought to be a safe procedure at experienced centers, even in patients with severe PH (5). In a retrospective and prospective evaluation of serious adverse events related to right heart catheter procedures in patients with pulmonary hypertension, the overall serious adverse events was 1.1% (6). We describe a case of complicated RHC in which the pigtail catheter became entangled in the tricuspid valve leaflet and was successfully dislodged using a modified method after the classic methods failed.

## CASE SUMMARIES

A 36-year old male patient with a 3-year history of unexplained cough and dyspnea was referred to our center for RHC. Initial echocardiographic evaluation revealed normal left ventricular size and function, D-shaped interventricular septum, severe Right Ventricular (RV) enlargement and RV dysfunction, mild tricuspid regurgitation, with Pulmonary Artery Pressure (PAP) of 80 mmHg. Pulmonary CT angiography was suggestive of CTEPH.

**Figure 1.**
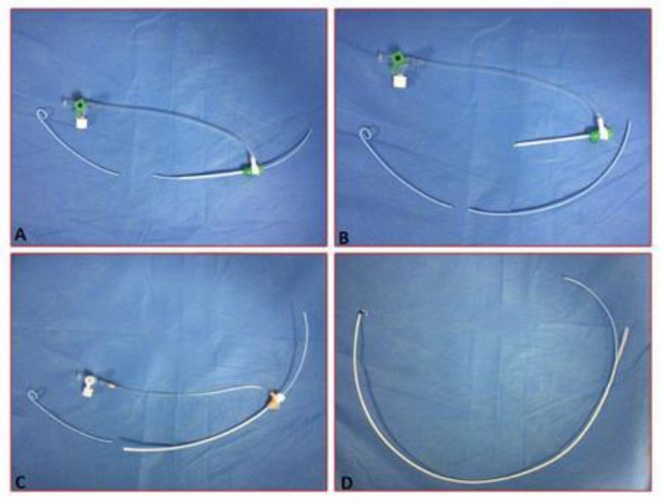
During right heart catheterization, the 6F pigtail catheter (arrow) was entrapped in the chordate tendineae of the septal leaflet of the tricuspid valve (A). The introducing sheath was removed after the hub of the pigtail catheter was cut (B). A biopsy sheath was introduced over the catheter (C). The sheath was peeled off until the cut end of the pigtail appeared (D).

**Figure 2.**
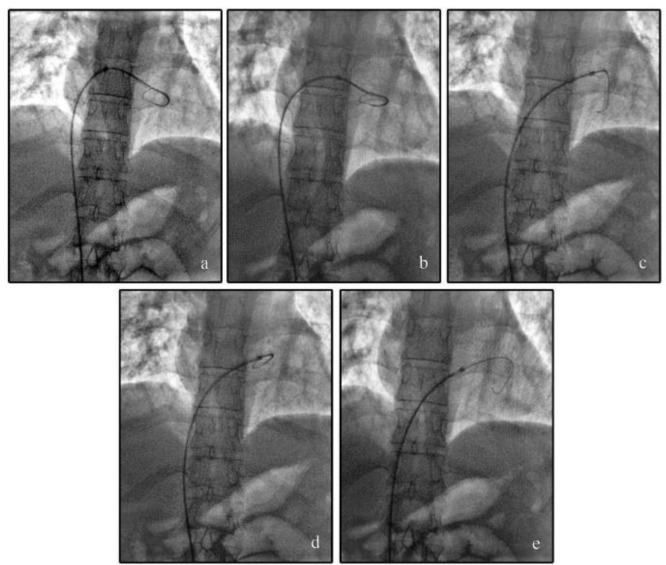
Angiographic views of the procedure: Entanglement of the 6F pigtail catheter (arrow) (a,b). Covering the catheter by forward pushing the sheet and removal of the pigtail catheter (c,d,e).

To perform a classic pulmonary catheterization we inserted a 6 F, 100 cm pigtail catheter (Cordis corporation, Miami, FL, USA) through a 6 F sheath introducer from the right femoral vein and passed it over a 0.035 inch, 150 cm steel tip-deflecting wire (Merit Medical systems, Utah, USA) into the RV. After advancing the catheter into the RV Outflow Tract (RVOT), retraction was attempted but it was unsuccessful. A bedside transthoracic echocardiography revealed entrapment of the pigtail catheter in the chordate tendineae of the septal leaflet of the tricuspid valve. We employed several methods, such as using a guide wire to straighten the pigtail catheter, and gentle clockwise and counterclockwise rotation of the catheter. But these manipulations all failed to release the catheter. Then we inserted a 0.035 inch guide wire from its hard tip into the pigtail catheter to remove the entrapped catheter but it was unsuccessful. Before transferring the patient to the Operating Room (OR), we attempted to try a different approach and push the chordae away instead of pulling the catheter back. To do so, we decided to advance a long sheath over the pigtail catheter. First, the sheath introducer was removed (Figure 1A). Then, the hub of the pigtail catheter was cut (Figure 1B) and a 7 F; 90 cm biopsy sheath (Cordis Corporation, Miami, FL, USA) which had the hemostatic valve cut off was advanced over the pigtail catheter with the support of the exchange guide wire (Figure 1C). After introducing the sheath for several centimeters, the pigtail catheter bended into the RV and further advancement of the sheath over the pigtail catheter became impossible. So we started shortening the sheath by cutting it gradually, until the end of the pigtail appeared (Figure 1D). Then the end of the pigtail was pulled back manually using a forceps preventing further bending and the sheath was introduced over the pigtail like an over the wire angioplasty balloon. The sheath advanced over the pigtail and pushed the chordate away, so the whole system was withdrawn easily. After the procedure the patient underwent echocardiography which showed no complication (Figure 2).

## DISCUSSION

RHC is a relatively safe procedure with low morbidity and mortality rates in experienced centers (6). Pigtail catheters are usually employed in RHC. Technical complications including knotting, hooking and entrapment of these catheters in intra-cardiac structures and valves have been rarely encountered (7,8). Knotting of an intravascular catheter was first reported by Johansson et al. in 1954 (6).

Karanikas et al. analyzed 113 cases of knotted intravascular devices/catheters. Open cardiotomy was required in five cases, in three the intracardiac knot involved the chordae tendineae of the tricuspid valve (9). In most cases, gentle manipulation of the catheter resulted in successful retrieval of the catheter. However, caution must be taken as forceful traction may result in papillary muscle, chordal and tricuspid valve rupture, necessitating urgent operative management (10,11) and one should bear in mind that cardiac surgery may be associated with poor outcomes in patients with preoperative PH (12,13). In our case the pigtail catheter was entrapped in chordae tendineae and gentle rotation and traction maneuvers failed to retrieve the catheter. In this technique, instead of manipulating the catheter via its internal lumen, the catheter was used as a rail for another bigger catheter. By introducing a bigger sheath over the catheter and fitting it with shortening at the end with cutting, we were able to remove the instrument. On pulling the entrapped catheter resistance can be felt. Also acute angulation of the catheter on fluoroscopy suggests entrapment. Transthoracic Echocardiogram (TTE) is an important tool, which shows the exact site of entrapment which is usually within the chordae tendineae of the septal leaflet of the tricuspid valve. Entrapments in other right heart structures were also reported. Device entrapment within Chiari’s network has been reported. So we employed a modified technique described before to release the entrapped catheter (14). This technique does not require any adjustment for the patients’ height or catheter’s length and tend to be a safe and inexpensive method when such complications are encountered and should be considered before transferring the patient to the operating room.

## CONCLUSION

RHC is an invasive method for evaluation of patients with elevated pulmonary artery hypertension. It is considered a safe method even though serious complications have been reported. One of the most feared complications is catheter entrapment. In most cases retrieving the catheter with simple maneuvers is possible but if it is unsuccessful our method could be used before referring the patient for surgery.
